# MVA-based vaccine candidates expressing SARS-CoV-2 prefusion-stabilized spike proteins of the Wuhan, Beta or Omicron BA.1 variants protect transgenic K18-hACE2 mice against Omicron infection and elicit robust and broad specific humoral and cellular immune responses

**DOI:** 10.3389/fimmu.2024.1420304

**Published:** 2024-08-29

**Authors:** Patricia Pérez, David Astorgano, Guillermo Albericio, Sara Flores, Cristina Sánchez-Corzo, María A. Noriega, Pedro J. Sánchez-Cordón, Nuria Labiod, Rafael Delgado, José M. Casasnovas, Mariano Esteban, Juan García-Arriaza

**Affiliations:** ^1^ Department of Molecular and Cellular Biology, Centro Nacional de Biotecnología (CNB), Consejo Superior de Investigaciones Científicas (CSIC), Madrid, Spain; ^2^ Centro de Investigación Biomédica en Red de Enfermedades Infecciosas (CIBERINFEC), Madrid, Spain; ^3^ Pathology Department, Centro de Investigación en Sanidad Animal (CISA), Instituto Nacional de Investigación y Tecnología Agraria y Alimentaria (INIA), Consejo Superior de Investigaciones Científicas (CSIC), Madrid, Spain; ^4^ Instituto de Investigación Hospital Universitario 12 de Octubre (imas12), Madrid, Spain; ^5^ Department of Medicine, School of Medicine, Universidad Complutense de Madrid, Madrid, Spain; ^6^ Department of Macromolecular Structures, Centro Nacional de Biotecnología (CNB), Consejo Superior de Investigaciones Científicas (CSIC), Madrid, Spain

**Keywords:** COVID-19, SARS-CoV-2, MVA-based vaccine, variants of concern, S protein, immunogenicity, efficacy, mice

## Abstract

Despite the decrease in mortality and morbidity due to SARS-CoV-2 infection, the incidence of infections due to Omicron subvariants of SARS-CoV-2 remains high. The mutations acquired by these subvariants, mainly concentrated in the receptor-binding domain (RBD), have caused a shift in infectivity and transmissibility, leading to a loss of effectiveness of the first authorized COVID-19 vaccines, among other reasons, by neutralizing antibody evasion. Hence, the generation of new vaccine candidates adapted to Omicron subvariants is of special interest in an effort to overcome this immune evasion. Here, an optimized COVID-19 vaccine candidate, termed MVA-S(3P_BA.1), was developed using a modified vaccinia virus Ankara (MVA) vector expressing a full-length prefusion-stabilized SARS-CoV-2 spike (S) protein from the Omicron BA.1 variant. The immunogenicity and efficacy induced by MVA-S(3P_BA.1) were evaluated in mice in a head-to-head comparison with the previously generated vaccine candidates MVA-S(3P) and MVA-S(3Pbeta), which express prefusion-stabilized S proteins from Wuhan strain and Beta variant, respectively, and with a bivalent vaccine candidate composed of a combination of MVA-S(3P) and MVA-S(3P_BA.1). The results showed that all four vaccine candidates elicited, after a single intramuscular dose, protection of transgenic K18-hACE2 mice challenged with SARS-CoV-2 Omicron BA.1, reducing viral loads, histopathological lesions, and levels of proinflammatory cytokines in the lungs. They also elicited anti-S IgG and neutralizing antibodies against various Omicron subvariants, with MVA-S(3P_BA.1) and the bivalent vaccine candidate inducing higher titers. Additionally, an intranasal immunization in C57BL/6 mice with all four vaccine candidates induced systemic and mucosal S-specific CD4^+^ and CD8^+^ T-cell and humoral immune responses, and the bivalent vaccine candidate induced broader immune responses, eliciting antibodies against the ancestral Wuhan strain and different Omicron subvariants. These results highlight the use of MVA as a potent and adaptable vaccine vector against new emerging SARS-CoV-2 variants, as well as the promising feature of combining multivalent MVA vaccine candidates.

## Introduction

The COVID-19 pandemic has been one of the largest outbreaks caused by an infectious agent in the modern era. Since the first cases, the causal agent, the SARS-CoV-2 coronavirus, has evolved giving rise to several variants of concern (VoCs) that have emerged during the pandemic. In particular, the SARS-CoV-2 Omicron (B.1.1.529) VoC was first identified in South Africa in November 2021 and spread rapidly throughout the world (1). Several Omicron subvariants have been discovered and new ones continue to emerge, with the ‘standard’ sublineage being termed BA.1 (or B.1.1.529.1). Compared to the ancestral Wuhan strain and other VoCs, the Omicron variants have the highest number of mutations throughout the genome. For example, the Omicron spike (S) protein has acquired at least 30 amino acid substitutions, 3 in-frame deletions, and a 3-amino acid insertion. Many of these mutations (>15) accumulate in the receptor-binding domain (RBD), which changes the interaction between the S protein and its receptor, angiotensin-converting enzyme 2 (ACE2), increasing infectivity and transmissibility (2, 3).

The emergence of the Omicron variant posed a challenge to the pre-existing immunity generated by both vaccination and natural infection (4, 5). The currently approved COVID-19 vaccines can protect against morbidity and mortality, but the molecular changes experienced by the S protein have increased the immune escape already observed in prior waves with previous VoCs (6). Several studies highlight the reduction of neutralizing antibody titers against Omicron and the importance of booster doses to maintain neutralization levels (7). Thus, despite the proven effectiveness of the COVID-19 vaccines authorized for humans and the global vaccination campaign, the emergence of SARS-CoV-2 VoCs, such as the Omicron (B.1.1.529), and the continuous appearance of mutations threatens the process of generating an effective and protective vaccine, leading to the need for updated vaccines and the use of multivalent regimens (6).

We have previously described that modified vaccinia virus Ankara (MVA) vectors expressing a native full-length Wuhan-derived SARS-CoV-2 S protein (termed MVA-S) or a prefusion-stabilized S protein containing 3 proline (3P) substitutions in the S2 region (A942P, K986P, and V987P) and the furin cleavage site mutated to avoid the processing of S into the S1 and S2 regions and fusion activation (termed MVA-S(3P)), were highly immunogenic and effective in mice (8–15), hamsters (16, 17), or rhesus macaques (18), with MVA-S(3P) being superior in S protein expression levels, immunogenicity and efficacy to MVA-S. Furthermore, we also recently demonstrated that an MVA vector analogous to MVA-S(3P) but adapted to the SARS-CoV-2 Beta (B.1.351) VoC, termed MVA-S(3Pbeta), elicited in immunized mice potent systemic and local T-cellular and humoral immune responses against the ancestral SARS-CoV-2 Wuhan strain and Beta VoC, as well as cross-neutralizing antibodies against other VoCs, and conferred protection to K18-hACE2 mice against a lethal challenge with Beta VoC (12).

To adapt our vaccine candidate to the Omicron SARS-CoV-2 landscape, we generated and characterized an MVA-based vaccine candidate expressing an optimized full-length prefusion-stabilized S protein, also containing 3 proline (3P) substitutions in the S2 region, derived from SARS-CoV-2 Omicron BA.1, termed MVA-S(3P_BA.1). Preclinical evaluation of MVA-S(3P_BA.1) in head-to-head comparison with previously generated MVA-S(3P) and MVA-S(3Pbeta), which share the same prefusion-stabilized S protein optimizations but from Wuhan strain and Beta variant, respectively, and evaluation of a bivalent vaccine candidate composed of a combination of MVA-S(3P) and MVA-S(3P_BA.1) demonstrated that an intramuscular (IM) dose of all four vaccine candidates protected transgenic K18-hACE2 mice against Omicron BA.1 infection, reducing SARS-CoV-2 mRNA, infectious viral loads and the levels of proinflammatory cytokines in the lungs, with decreased lung histopathological lesions. Vaccination also elicited anti-S IgG and neutralizing antibodies against different VoCs with MVA-S(3P_BA.1) and the bivalent vaccine candidate inducing higher titers. Furthermore, all four vaccine candidates elicited, in C57BL/6 mice, systemic and mucosal SARS-CoV-2 Omicron S-specific CD4^+^ and CD8^+^ T-cell and humoral immune responses after a single intranasal (IN) immunization. In particular, the bivalent vaccine candidate demonstrated advantage in generating broader immune responses, inducing the production of antibodies against the ancestral Wuhan strain, Omicron BA.1 and other subvariants.

## Materials and methods

### Animals and ethics statement

Female mice of the transgenic K18-hACE2 line (6–8 weeks old), which express the human ACE2 receptor, were acquired from the Jackson Laboratory [034860-B6.Cg-Tg(K18-ACE2)2Prlmn/J, genetic background C57BL/6J x SJL/J)F2], and efficacy experiments using these mice were performed in the biosafety level 3 (BSL-3) facilities at the Centro de Investigación en Sanidad Animal (CISA)-Instituto Nacional de Investigaciones Agrarias (INIA)-Consejo Superior de Investigaciones Científicas (CSIC) (Valdeolmos, Madrid, Spain). Female C57BL/6OlaHsd mice (6–8 weeks old) used for immunogenicity experiments were purchased from Envigo Laboratories and stored in the animal facility of the Centro Nacional de Biotecnología (CNB-CSIC) (Madrid, Spain). The Ethical Committees of Animal Experimentation (CEEA) of the CNB-CSIC, CISA-INIA-CSIC and the Division of Animal Protection of the Comunidad de Madrid approved these animal studies (PROEX 49/20, 169.4/20 and 161.5/20). Animal procedures followed the international guidelines and Spanish law under the Royal Decree (RD) 53/2013.

### Cells

DF-1 cells (a spontaneously immortalized chicken embryo fibroblast [CEF] cell line; ATCC catalog number CRL-12203) and HeLa cells (human epithelial cervix adenocarcinoma; ATCC catalog number CCL-2) were cultured in Dulbecco’s modified Eagle’s medium (DMEM) (Gibco-Life Technologies) supplemented with penicillin (100 U/mL; Sigma-Aldrich), streptomycin (100 mg/mL; Sigma-Aldrich), L-glutamine (2 mM; Sigma-Aldrich), non-essential amino acids (0.1 mM; Sigma-Aldrich), amphotericin B (Fungizone, 0.5 mg/mL; Gibco-Life Technologies), gentamicin (50 mg/mL; Sigma-Aldrich) and 10% heat-inactivated fetal bovine serum (FBS) (Gibco-Life Technologies). Vero-E6 cells (from African green monkey kidney, ATCC catalog number CRL-1586) were grown in DMEM (Gibco-Life Technologies) supplemented with HEPES (10 mM; Gibco-Life Technologies), non-essential amino acids (0.1 mM; Sigma-Aldrich), penicillin (100 U/mL; Sigma-Aldrich), streptomycin (100 mg/mL; Sigma-Aldrich), and 10% heat inactivated FBS. Vero/TMPRSS2 (Vero-E6 cell line modified to constitutively express TMPRSS2 serine protease, under geneticin selection, in order to be highly susceptible to SARS-CoV-2 infection) were maintained in DMEM (Gibco-Life Technologies) supplemented with HEPES (10 mM; Gibco-Life Technologies), nonessential amino acids (0.1 mM; Sigma-Aldrich), penicillin (100 U/mL; Sigma-Aldrich), streptomycin (100 mg/mL; Sigma-Aldrich), Geneticin (G418, 1 mg/mL, Merck-Life Sciences), and 10% heat inactivated FBS. Cell cultures were maintained at 37°C in a humidified incubator containing 5% CO_2_.

### Viruses

We used the attenuated MVA wild-type (MVA-WT) poxvirus strain, derived from the Chorioallantois vaccinia virus Ankara strain (19), and the MVA-S(3P) and MVA-S(3Pbeta) vaccine candidates (12–14). MVA-WT was employed as the parental virus for the generation of the MVA-S(3P_BA.1) vaccine candidate expressing an Omicron BA.1 (B.1.1.529.1) derived human codon optimized full-length prefusion-stabilized SARS-CoV-2 S protein, containing three mutations in the furin cleavage site (R682G, R683S, and R685S) to prevent cleavage of the S protein in S1 and S2 domains, and 3 proline (3P) substitutions in the S2 region that stabilize the S protein in a prefusion conformation (A942P, K986P, and V987P). All MVA viruses were grown in permissive culture chicken cells (DF-1) to produce a master virus seed stock (passage 2 [P2] stock) and titrated in DF-1 cells using a plaque immunostaining assay, as previously described (20). For use as inoculum in animal experiments, MVA viruses were expanded in DF-1 cells and purified by centrifugation through two 36% (wt/vol) sucrose cushions in 10 mM Tris-HCl (pH 9). All viral stocks were free of contamination with mycoplasma (checked by Mycoplasma Gel Detection kit; Biotools), bacteria (checked by growth on LB plates without ampicillin), or fungi (checked by growth on Columbia blood agar plates; Oxoid).

The SARS-CoV-2 MAD6 isolate, similar to the Wuhan strain but containing the D614G mutation in the S protein (21), was kindly provided by Dr. José M. Honrubia and Prof. Luis Enjuanes (CNB-CSIC, Madrid, Spain). The SARS-CoV-2 B.1.1.529 Omicron variant (hCoV-19/Belgium/rega-20174/2021, EPI_ISL_6794907) was supplied by Prof. Piet Maes from KU Leuven (Belgium) through Dr. Robbert Boudewijns and Dr. Kai Dallmeier (KU Leuven, Belgium). The SARS-CoV-2 Omicron BA.5 (EPI_ISL_13424827) and BQ.1.1 (EPI_ISL_15653663) subvariants were provided by Prof. Rafael Delgado (Hospital Universitario 12 de Octubre, Madrid, Spain). SARS-CoV-2 viral stocks were amplified by propagation in Vero/TMPRSS2 cells by inoculation at a multiplicity of infection (MOI) of 0.001 plaque-forming units (PFUs)/cell (passage 2). Cell supernatants were harvested at 72 h post-infection (hpi), cleared by centrifugation, aliquoted, and stored at -80°C. Virus infectivity titers were determined by a standard plaque assay or by a median tissue culture infectious dose (TCID_50_) assay in Vero-E6 cells, as previously described (12–14).

### Construction of plasmid transfer vector pCyA-S(3P_BA.1) and generation of MVA-S(3P_BA.1) vaccine candidate

The plasmid transfer vector pCyA-S(3P_BA.1) was designed to generate the MVA-S(3P_BA.1) vaccine candidate. A full-length prefusion-stabilized SARS-CoV-2 Omicron BA.1 (B.1.1.529.1) S gene (GISAID accession ID: EPI_ISL_6794907) was inserted into the thymidine kinase (TK) locus of the parental virus MVA-WT, under the transcriptional control of the viral synthetic early/late (sE/L) promoter and with a Kozak sequence (GCCACC) before the ATG initiation codon of the S gene. The encoded full-length prefusion-stabilized SARS-CoV-2 Omicron BA.1 (B.1.1.529.1) S protein was human codon optimized and contains 3 mutations in the furin cleavage site (R682G, R683S, and R685S) to prevent cleavage of the S protein in S1 and S2 domains, and the same 3 proline (3P) prefusion-stabilizing amino acid mutations (A942P, K986P, and V987P) included in the MVA-S(3P) (14), and MVA-S(3Pbeta) (12) vaccine candidates validated previously. Briefly, a 3,813-kbp DNA fragment encoding the SARS-CoV-2 Omicron BA.1 (B.1.1.529.1) full-length prefusion-stabilized S gene was synthesized by GeneArt (Thermo Fisher Scientific) and inserted into plasmid vector pCyA (22), obtaining the plasmid transfer vector pCyA-S(3P_BA.1) (11,313 bp). This plasmid includes a β-galactosidase (β-Gal) reporter gene between two repetitions of the left TK-flanking arm, which allows the deletion by homologous recombination of the reporter from the final recombinant virus after successive passages.

Cultured DF-1 cells (3 × 10^6^ cells) were infected with parental MVA-WT virus at a MOI of 0.02 PFUs/cell and transfected 1 h later with 10 μg of pCyA-S(3P_BA.1) plasmid, using Lipofectamine 2000 (Invitrogen) reagent, according to the manufacturer’s recommendations. At 72 hpi, cells were harvested, lysed by freeze–thaw cycling, sonicated, and used for recombinant virus screening. Recombinant MVA-S(3P_BA.1) viruses containing the SARS-CoV-2 Omicron BA.1 (B.1.1.529.1) full-length prefusion-stabilized S gene, inserted in the TK locus, and transiently coexpressing the β-Gal marker gene were selected by three consecutive rounds of plaque purification in DF-1 cells stained with X-Gal (5-bromo-4-chloro-3-indolyl-β-D-galactopyranoside, 1.2 mg/mL) (Sigma-Aldrich). In subsequent plaque purification steps, recombinant MVA-S(3P_BA.1) viruses with the β-Gal gene deleted by homologous recombination between the left TK arm and the short-left TK arm repeat flanking the marker were isolated by three additional consecutive rounds of plaque purification screening for non-staining viral foci in DF-1 cells in the presence of X-Gal (1.2 mg/mL). In each round of plaque purification, the isolated plaques were grown in DF-1 cells, and the crude viruses obtained were used for the next round of plaque purification. The resulting recombinant virus MVA-S(3P_BA.1) was grown, purified and titrated as previously described (14).

### Expression of SARS-CoV-2 S protein by Western blotting

To check the correct expression of SARS-CoV-2 prefusion-stabilized S protein by MVA-S(3P_BA.1) vaccine candidate, monolayers of human HeLa cells grown in 24-well plates were infected at 5 PFUs/cell with MVA-S(3P_BA.1), MVA-S(3P), or with control virus MVA-WT in the presence or absence of tunicamycin (10 μg/mL, Sigma-Aldrich), an inhibitor of N-glycosylation. At different times post-infection (4, 7 or 24 hpi), equal amounts of cell extracts were lysed under reducing (in the presence of 1X Laemmli plus β-mercaptoethanol; Sigma-Aldrich) or nonreducing conditions (in the presence of 1X Laemmli without β-mercaptoethanol; Sigma-Aldrich), fractionated by 7% sodium dodecyl sulfate-polyacrylamide gel electrophoresis (SDS-PAGE), and analyzed by Western blotting with a rabbit polyclonal anti-SARS-CoV-2 RBD antibody (Genetex; diluted 1:1,000) to analyze the expression of the SARS-CoV-2 S protein. For a viral loading control, we used a rabbit anti-vaccinia virus (VACV) E3 antibody (CNB-CSIC, diluted 1:1,000). An anti-rabbit horseradish peroxidase (HRP)-conjugated antibody (Sigma-Aldrich; diluted 1:5,000) was used as the secondary antibody. SARS-CoV-2 S protein expression stability of MVA-S(3P_BA.1) vaccine candidate was also analyzed by Western Blotting after 9 consecutive passages, as previously reported (14).

### Analysis of virus growth

To study the virus growth profile of MVA-S(3P_BA.1) in comparison to that of MVA-S(3P) and MVA-WT, monolayers of permissive DF-1 cells were infected at 0.01 PFUs/cell with MVA-S(3P_BA.1), MVA-S(3P) or MVA-WT, harvested at different times post-infection (0, 24, 48, and 72 hpi), and virus titers in cell lysates were determined by a plaque immunostaining assay, as previously described (9).

### Efficacy study schedule in K18-hACE2 transgenic mice

Groups of female K18-hACE2 mice (n=5/group; 6-8 weeks-old at the beginning of the study) immunized with one IM dose (1 x 10^7^ PFUs in 100 μL of PBS (50 μL/leg)) of MVA-S(3P), MVA-S(3Pbeta), MVA-S(3P_BA.1), or a bivalent vaccine candidate composed of a 1:1 mixture of MVA-S(3P) and MVA-S(3P_BA.1) were used to evaluate the efficacy of the vaccine candidates. Mice inoculated with non-recombinant MVA-WT were used as a control group. On day 14 post-immunization, blood was collected from each mouse by submandibular bleeding. The blood was incubated at 37 °C for 1 h, maintained at 4 °C overnight, and centrifuged at 3,600 rpm for 20 min at 4 °C to obtain serum samples. The obtained serum samples were then inactivated at 56°C for 30 min and stored at -20°C until analysis of humoral immune responses. Five weeks after the immunization (day 35), all mice were anesthetized in an isoflurane chamber and challenged with 1 x 10^5^ PFUs of SARS-CoV-2 Omicron (B.1.1.529.1) BA.1 strain by the IN route in 50 μL of PBS. Mice were then monitored for body weight changes, signs and symptoms of disease, and mortality for 6 days after the challenge. At day 6 post-challenge (day 41), mice were euthanized, and lung, nasal wash (NW), and serum samples were collected. The entire left lung lobe was removed from each mouse and immersion-fixed in zinc formalin (Sigma-Aldrich) for 48 h. After the fixation period, samples were routinely processed and embedded in paraffin for subsequent histopathological evaluation. The right lung lobes were divided longitudinally into two, with one part placed in RNALater stabilization reagent (Sigma-Aldrich) and stored at -80°C until RNA extraction, and the other lung part was weighed and stored at -80°C until analysis of virus yields. NW samples from each mouse were collected by flushing into the nasal cavity 400 μL of PBS; then, the samples were spun down to separate cellular pellet and supernatant and stored at -80°C until use for RNA extraction and detection of infectious virus, respectively.

### Analysis of viral yields by plaque assay

Lung and NW samples from K18-hACE2 mice, harvested at day 6 post-challenge, were analyzed for the presence of SARS-CoV-2 infectious virus using a plaque assay, as previously described (12–14). Lungs were harvested, weighed, and stored directly at -80°C until homogenization with a gentleMACS dissociator (Miltenyi Biotec) in 2 mL of PBS buffer. Undiluted and serial ten-fold dilutions of homogenized lung tissue or NW samples were added in triplicate to Vero-E6 cell monolayers seeded in 12-well plates at 5 x 10^5^ cells/well. After 1 h of adsorption, the inoculum was removed and plates were incubated at 37°C, 5% CO_2_ in 2:1 DMEM 2X-4% FBS: Avicel^®^ RC-591 (microcrystalline cellulose and carboxymethylcellulose sodium, DuPont Nutrition Biosciences ApS). After 3 days, cells were fixed for 1 h with 10% formaldehyde (Sigma-Aldrich), the supernatant was removed, and plaques were visualized by adding 0.5% crystal violet (Sigma-Aldrich). SARS-CoV-2 titers were determined in PFUs per gram of lung tissue or in PFUs per mL of NW.

### Quantification of SARS-CoV-2 and cytokine mRNA by reverse transcription-quantitative polymerase chain reaction (RT-qPCR)

At day 6 post-challenge, lung and NW samples from K18-hACE2 mice were harvested and mRNA from these samples was extracted, as previously described (12–14). Lungs, stored in RNALater (Sigma-Aldrich) at -80°C, were homogenized using a gentleMACS dissociator (Miltenyi Biotec) in 2 mL of RLT buffer (Qiagen) containing β-mercaptoethanol (Sigma-Aldrich). A total of 600 μL of homogenized lung tissue was utilized to isolate total RNA using the RNeasy Mini Kit (Qiagen), according to the manufacturer’s specifications. On the other hand, cellular pellets of NW samples were resuspended in 50 μL of RLT buffer (Qiagen) containing β-mercaptoethanol (Sigma-Aldrich) and used to extract RNA using the RNeasy Mini Kit (Qiagen), according to the manufacturer’s specifications.

RNA samples from lung and NW were subjected to analysis for quantifying SARS-CoV-2 mRNA using RT-qPCR, following a previously described method (12–14). SARS-CoV-2 viral mRNA content was determined using previously validated sets of primers and probes specific for the SARS-CoV-2 subgenomic RNA for protein E and the genomic RNA-dependent RNA polymerase (RdRp) (23) and the conservative region in ORF1 (24). Gene expression was normalized to the expression of the cellular 28S ribosomal RNA gene (23). Additionally, the mRNA expression levels of key proinflammatory cytokines (IL-6, IL-10, CCL2, CCL11, IFNγ, TNFα and CXCL10) were also measured in lung samples using specific TaqMan probes (Thermo Fisher Scientific; the sequence will be provided upon request). The specific gene expression was also calculated relative to the expression of the cellular 28S ribosomal RNA gene, as previously described (12–14). mRNA arbitrary units (A.U.) were quantified relative to negative RNA samples (from uninfected mice) using the 2^-ΔΔCt^ method. All samples were tested in duplicates.

### Lung histopathology

Lung histopathology was performed as previously described (12–14). Histopathological evaluations were performed by a single veterinary pathologist (Pedro J. Sánchez-Cordón, CISA-INIA-CSIC) who was blinded to the identity or group of each mouse. To assess the character and severity of lung histopathological lesions, lung inflammation scoring parameters based on previous reports on SARS-CoV-2 infection in mouse models were used (25). These histopathological parameters were graded following a semi-quantitative scoring system as follows: (0) no lesion; (1) minimal lesion; (2) mild lesion; (3) moderate lesion; (4) severe lesion. The cumulative scores of the histopathological lesions provided the total score for each animal. In each experimental group, individual scores were used to calculate the group average. In addition, haematoxylin & eosin-stained sections were visually scored 0–6 based on the percentage of lung area affected by inflammatory lesions as follows: 0% lung injury (score 0); < 5% (score 1); 6-10% (score 2); 11–20% (score 3); 21–30% (score 4); 31–40% (score 5); > 40% (score 6). In each experimental group, individual scores were used to calculate the group average.

### Enzyme-linked immunosorbent assay (ELISA)

The titers of anti-S IgG antibodies in individual or pooled sera from immunized mice were measured by ELISA, as previously described (12–14). Endpoint IgG titers were measured as the last serum dilution that gave an absorbance value at 450 nm at least three times higher than that of a naive serum. Moreover, the levels of anti-S IgA ang IgG antibodies in pooled bronchoalveolar (BAL) samples were also measured by ELISA, and the area under the curve (AUC) was calculated using the GraphPad Prism version 10.2.1 Software. The soluble SARS-CoV-2 S proteins used to coat the plates were derived from the Wuhan strain (GenBank accession number MN908947.3), and the Omicron BA.1 (OL672836.1), BA.5 (ON249995.1) and XBB.1.5 (OP790748.1) subvariants. The recombinant expression vectors for protein production were prepared as previously described (9, 12–14), and the soluble S proteins were expressed in mammalian cells and purified from cell supernatants as previously reported (9).

### Neutralization of live SARS-CoV-2 or pseudotyped variants of concern

The capacity of individual or pooled sera obtained from immunized mice to neutralize live SARS-CoV-2 virus was measured using a microneutralization test (MNT) assay in a BSL-3 laboratory at the CNB-CSIC, as previously described (12–14). Serially diluted serum samples in DMEM-2% FBS medium were incubated at a 1:1 ratio with 100 TCID_50_ of SARS-CoV-2 parental Wuhan strain virus (MAD6 isolate, containing the D614G mutation in the S protein), Omicron BA.1, BA.5 or BQ.1.1 subvariants in 96-well tissue culture plates for 1 h at 37°C. Then, mixtures of serum samples and SARS-CoV-2 were added in triplicate to Vero-E6 cell monolayers seeded in 96-well plates at 1.5 x 10^4^ cells/well. Cells were allowed to be infected for 72 h, after which they were fixed and subsequently stained with 5% Crystal Violet (Sigma-Aldrich). Once the plates were dry, cell monolayers were solubilized with 1% SDS (Sigma-Aldrich) and absorbance was measured at 570 nm to determine the neutralization percentage. On the other hand, SARS-CoV-2 pseudotyped vesicular stomatitis virus (VSV) expressing the SARS-CoV-2 S protein from different variants was also used to evaluate the neutralization capacity and to indirectly test the breadth of neutralization capacity of pooled serum samples obtained from immunized mice, as previously described (11, 12, 16). The SARS-CoV-2 S variants used were S_614G, Omicron BA.1 (GISAID: EPI_ISL_6794907), BA.4/BA.5 (GISAID: EPI_ISL_13424827), and BQ.1.1 (GISAID: EPI_ISL_15653663), produced as described elsewhere (26).

To obtain the 50% neutralization titers (NT_50_), half maximal effective concentration (EC_50_) and 95% confidence intervals (95% CI) were calculated using a nonlinear regression model fit with settings for agonist concentration versus normalized response curve using GraphPad Prism version 10.2.1 Software.

### Immunogenicity study schedule in C57BL/6 mice

To evaluate the immunogenicity of the MVA-based vaccine candidates against COVID-19, groups of female C57BL/6 mice (n=5/group; 6 to 8 weeks-old) were slightly anesthetized with isoflurane (1-chloro-2,2,2-trifluoroethyl difluoromethyl ether; Isoflo^®^, Zoetis Belgium SA) and each mouse received one dose of 1 x 10^7^ PFUs of MVA-S(3P), MVA-S(3Pbeta), MVA-S(3P_BA.1), or bivalent vaccine candidate composed of a 1:1 mixture of MVA-S(3P) and MVA-S(3P_BA.1) by the IN route in 50 μl of PBS. Mice inoculated with non-recombinant MVA-WT were used as a control group. No adverse effects were detected in immunized mice. Then, 14 days after the immunization, mice were euthanized by using a lethal dose of 10% xylazine (Xilagesic 20 mg/mL; Laboratorios Calier) + 10% ketamine (Imalgene 100 mg/mL; Merial Laboratorios) and spleens and BAL samples were obtained. Spleens extracted from each mouse were pooled per group, processed mechanically, blood-cell depleted, and filtered through 40-µm cell strainers until single-cell samples were obtained. Each mouse was insufflated twice with PBS 1X complemented with a protease inhibitor cocktail (Roche) to obtain BAL samples, which were centrifuged at 1,500 rpm for 5 min at 4°C to separate cellular pellets from supernatants. Single-cell samples from spleens along with BAL cells were used to measure the SARS-CoV-2 S-specific T-cell immune responses by an intracellular cytokine staining (ICS) assay, as previously described (9). Blood from each mouse was collected by cardiac puncture, maintained at 37°C for 1 h, kept at 4°C overnight, and centrifuged at 3,600 rpm for 20 min at 4°C to obtain serum samples. Serum samples and BAL supernatants were stored at -20°C until used, to analyze SARS-CoV-2-specific humoral immune responses.

### ICS assay

The magnitude, polyfunctionality and memory phenotype of SARS-CoV-2 S-specific CD4^+^ and CD8^+^ T cells expressing CD107a, and/or secreting IFNγ, and/or TNFα, and/or IL-2 were analyzed by an ICS assay, as previously described (9), in cells (splenocytes or BAL cells) stimulated with two SARS-CoV-2 S Omicron (B.1.1.529) peptide pools (1 µg/mL; JPT Peptide Technologies), spanning the S1 and S2 regions as consecutive 15-mers overlapping by 11 amino acids of the S protein from the Omicron (B.1.1.529) variant. Cells were acquired with a Gallios flow cytometer (Beckman Coulter), and analyses of the data were performed with the FlowJo software version 10.4.2 (Tree Star), as previously described (9).

### Statistical procedures

All graphs, calculations, and statistical analyses were performed using GraphPad Prism software version 10.2.1 (GraphPad Software). To determine differences between groups, ordinary one-way ANOVA of transformed data followed by Tukey’s multiple comparisons test was used for the statistical analysis of SARS-CoV-2 viral yields and SARS-CoV-2 and cytokine mRNA levels. An unpaired nonparametric Mann-Whitney test was employed for the statistical evaluation of lung histopathological scores and an ordinary one-way ANOVA followed by Tukey’s multiple comparisons test for the percentage of lung area with lesions. An unpaired ordinary one-way ANOVA followed by Tukey’s multiple comparisons test of transformed data was used for the statistical analysis of the IgG titers and the live virus NT_50_ neutralizing antibody titers, an unpaired ordinary one-way ANOVA followed by Tukey’s multiple comparisons test for AUC values of pooled BAL optical density units at 450 nm, and a Brown-Forsythe and Welch ANOVA test of transformed data for NT_50_ neutralizing antibody titers of pooled mouse serum samples against SARS-CoV-2 pseudotyped VSVs. Statistical analysis of the ICS assay data was realized as previously described (27), using an approach that corrects measurements for background response, calculating confidence intervals and p-values. Statistical significance is indicated as follows: *p < 0.033; **p < 0.002; ***p < 0.0002; ****p<0.0001.

## Results

### Generation of an MVA-based vaccine candidate expressing a prefusion-stabilized S protein from the SARS-CoV-2 Omicron BA.1 variant

**Figure 1:**
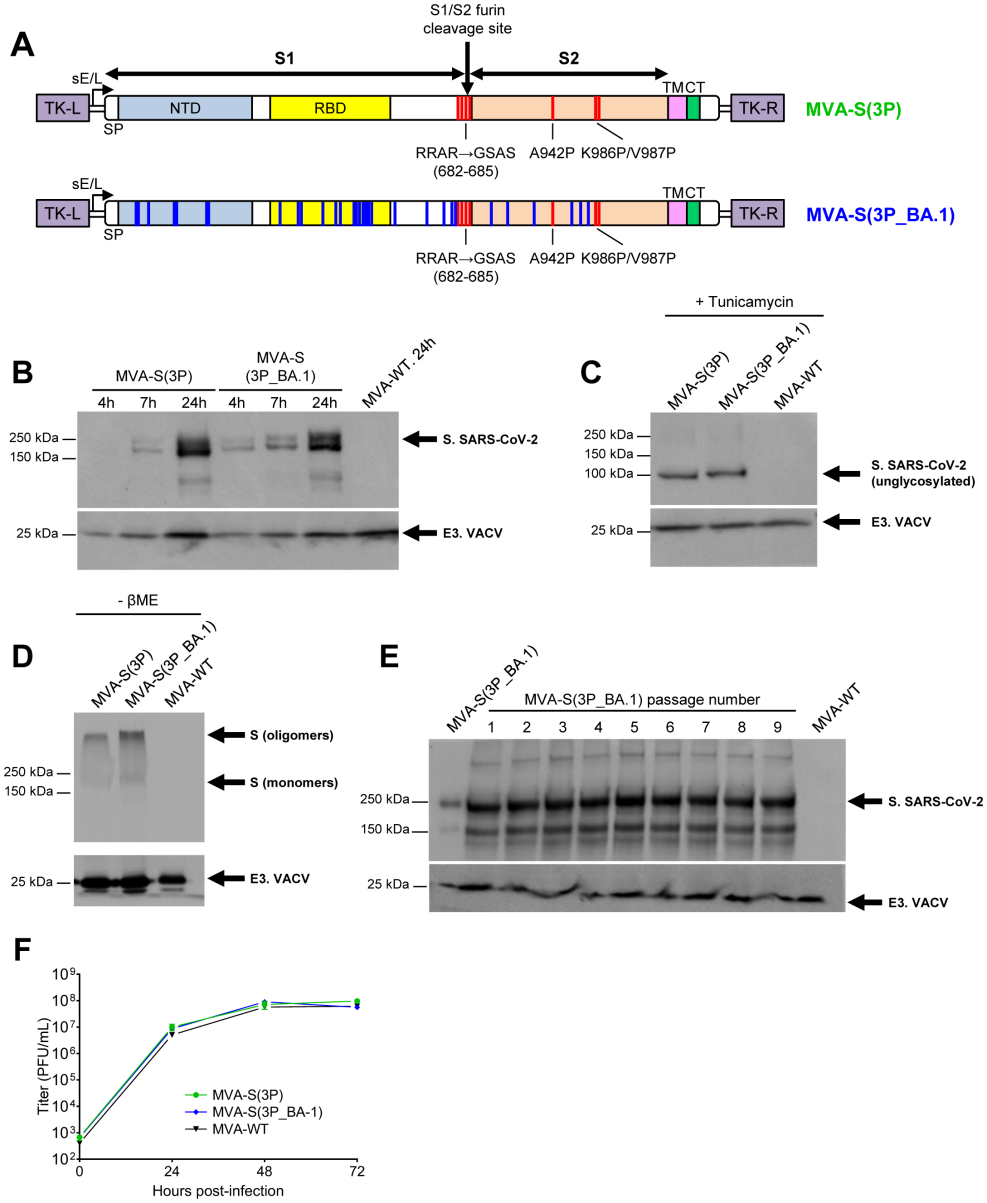
Design, generation, and *in vitro* characterization of MVA-S(3P_BA.1) vaccine candidate. **(A)** Scheme of the full-length prefusion-stabilized S proteins inserted in the MVA genome to generate the MVA-S(3P) and MVA-S(3P_BA.1) vaccine candidates. S1 and S2 regions are indicated, together with the amino acid mutations in the furin cleavage site and changes to prolines in the S2 region (indicated in red). The amino acid mutations derived from the Omicron (B.1.1.529) BA.1 variant are indicated in blue. The SARS-CoV-2 S gene is inserted within the TK locus of the MVA-WT virus and is driven by the sE/L virus promoter. SP: signal peptide; NTD: N-terminal domain; RBD: receptor- binding domain; TM: transmembrane; CT: cytoplasmic tail; TK-L: TK left; TK-R: TK right. **(B–E)** Expression of SARS-CoV-2 S protein by MVA-S(3P) and MVA-S(3P_BA.1) vaccine candidates. Rabbit polyclonal anti-S and anti-VACV E3 antibodies were used for protein identification on 7% SDS-PAGE. Size (in kilodaltons [kDa]) and migration of molecular weight markers are indicated. **(B)** Western blotting of MVA-infected (5 PFUs/cell) HeLa cell extracts at 4, 7 and 24 hpi, under reducing (plus β-mercaptoethanol) conditions. **(C)** Western blotting of MVA-infected (5 PFUs/cell) HeLa cell extracts at 24 hpi, treated with tunicamycin, and under reducing (plus β-mercaptoethanol) conditions. The detection of the unglycosylated S protein is indicated. **(D)** Western blotting of MVA-infected (5 PFUs/cell) HeLa cell extracts at 24 hpi, under nonreducing (-β-mercaptoethanol) conditions. The detection of S oligomers and monomers is indicated. **(E)** Genetic stability of MVA-S(3P_BA.1) vaccine candidate. Western blotting of DF-1 cell samples (24 hpi) infected with the master seed stock (P2 stock) of MVA-S(3P_BA.1) and with 9 successive passages at low MOI in DF-1 cells. Samples were analyzed under reducing (plus β-mercaptoethanol) conditions. **(F)** Viral growth kinetics of MVA-S(3P_BA.1). Monolayers of DF-1 cells were infected at 0.01 PFUs/cell with MVA-WT, MVA-S(3P) or MVA-S(3P_BA.1). At different times post-infection (0, 24, 48, and 72 hpi), virus titers (PFUs/mL) in cell lysates were quantified by a plaque immunostaining assay. The means of the results of two independent experiments are shown.

We have previously shown that MVA vectors expressing full-length prefusion-stabilized SARS-CoV-2 S proteins from Wuhan or Beta VoC were highly immunogenic and effective against virus infection in animal models. Now, with the global spread of the SARS-CoV-2 Omicron (B.1.1.529) variant and its subvariants, which dominate the community with the ability to escape the neutralization efficiency induced by prior vaccination or infections, we have generated a new MVA-based vaccine candidate targeting the Omicron BA.1 variant, termed MVA-S(3P_BA.1). We have selected BA.1 because it was the first widely distributed Omicron variant to appear, with many mutations common to all sub-lineages. MVA-S(3P_BA.1) expresses a full-length prefusion-stabilized S protein from the Omicron BA.1, which also contains three proline (3P) substitutions in the S2 region (A942P, K986P, and V987P) and lacks the furin cleavage site (**Figure 1A**). The S protein expression profile was characterized at different time points in non-permissive human HeLa cells infected with MVA-S(3P_BA.1) and MVA-S(3P), revealing a major 180-kDa protein product (**Figure 1B**). Furthermore, the S electrophoretic mobility reduction in the presence of tunicamycin indicated that the S protein was glycosylated (**Figure 1C**). In the absence of reducing agents, protein S exhibited electrophoretic migration consistent with the formation of oligomers, presumably trimers (**Figure 1D**). Analysis of SARS-CoV-2 S protein expression during 9 successive passages in permissive DF-1 cell cultures infected at low MOI (0.05 PFUs/cell) showed that MVA-S(3P_BA.1) efficiently expressed the S protein, demonstrating high expression stability (**Figure 1E**). Additionally, to analyze whether expression of the heterologous S protein affects MVA replication in cell culture, we evaluated the growth kinetics of MVA-S(3P_BA.1) compared with MVA-S(3P) and MVA-WT in permissive DF-1 cells. All three viruses had similar viral growth kinetics (**Figure 1F**), demonstrating that constitutive expression of the heterologous SARS-CoV-2 S protein does not weaken MVA vector replication under permissive conditions.

### Vaccination with MVA-S(3P), MVA-S(3Pbeta), MVA-S(3P_BA.1), or a bivalent MVA-S(3P)/MVA-S(3P_BA.1) combination protected K18-hACE2 transgenic mice against infection by SARS-CoV-2 Omicron BA.1 variant

**Figure 2:**
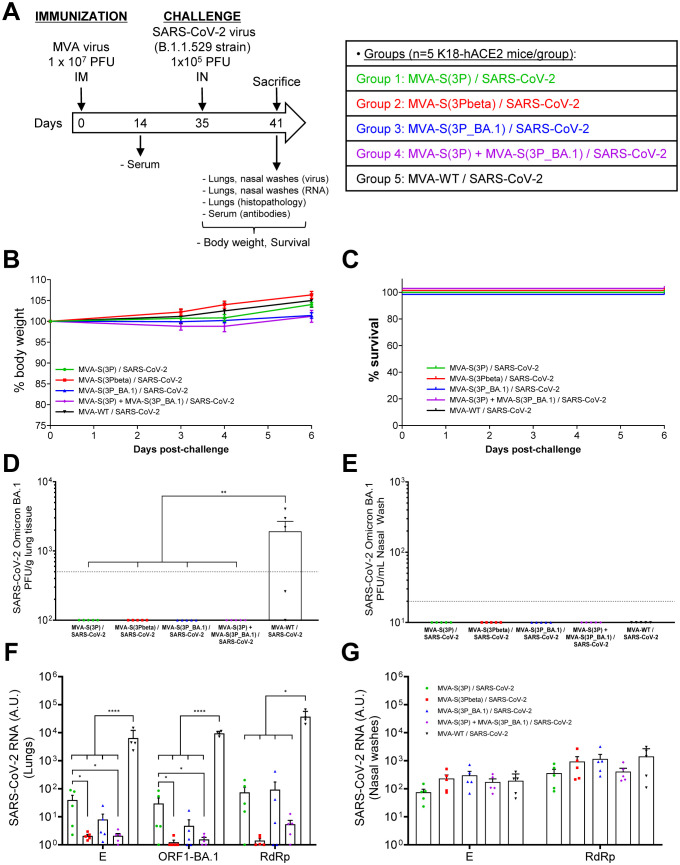
MVA-based vaccine candidates expressing S protein from different SARS-CoV-2 variants protect K18-hACE2 transgenic mice from SARS-CoV-2 Omicron (B.1.1.529) infection. **(A)** Efficacy schedule. Female K18-hACE2 transgenic mice (n=5 per group) were immunized by the IM route with one dose of 1 x 10^7^ PFUs of MVA-S(3P), MVA-S(3Pbeta), MVA-S(3P_BA.1), a bivalent vaccine candidate composed of a 1:1 combination of MVA-S(3P) and MVA-S(3P_BA.1) or MVA-WT (control group), as indicated. At day 14 post-immunization, serum samples were obtained from each mouse, as indicated. At day 35 (5 weeks post-immunization) mice were challenged intranasally with 1 x 10^5^ PFUs of SARS-CoV-2 Omicron (B.1.1.529) variant. At day 6 post-challenge, all mice were sacrificed and lungs, NW and serum samples collected as indicated. **(B, C)** The challenged mice were monitored for change of body weight **(B)** and mortality **(C)** for 6 days. **(D, E)** SARS-CoV-2 infectious virus in lung samples **(D)** and NW **(E)**. Detected by a plaque assay at 6 days after virus infection. Mean (PFUs/g of lung tissue or PFUs/mL of NW) from triplicates of each sample and the standard error of the mean (SEM) of each group are represented. The dashed line represents the limit of detection. **(F, G)** Virus replication in lung samples **(F)** and NW **(G)**. SARS-CoV-2 subgenomic E and genomic ORF1-BA.1 or RdRp mRNA detected by RT-qPCR at 6 days after virus infection. Mean RNA levels (in arbitrary units [A.U.] normalized to uninfected mice) from duplicates of each lung and NW sample and SEM of each group are represented. Ordinary one-way ANOVA of transformed data followed by Tukey’s multiple comparisons test: *p < 0.033; **p < 0.002; ****p<0.0001.

Next, we evaluated the protective efficacy of MVA-S(3P), its analogous MVA-S(3Pbeta) and MVA-S(3P_BA.1) or a bivalent vaccine candidate consisting of a 1:1 combination of MVA-S(3P) and MVA-S(3P_BA.1) in vaccinated K18-hACE2 mice infected with SARS-CoV-2 Omicron (B.1.1.529). Thus, female K18-hACE2 mice (n=5/group) were immunized intramuscularly with one dose (1 x 10^7^ PFUs/mouse) of MVA-S(3P), MVA-S(3Pbeta), MVA-S(3P_BA.1) or the bivalent MVA-S(3P)/MVA-S(3P_BA.1) vaccine candidate and then challenged, 5 weeks later, with an IN dose of SARS-CoV-2 Omicron (B.1.1.529) variant (1 x 10^5^ PFUs/mouse). Mice inoculated with MVA-WT were used as a control group of non-vaccinated infected mice (**Figure 2A**). Mice were supervised for changes in body weight and mortality for 6 days after SARS-CoV-2 infection. None of the mice experience body weight loss (**Figure 2B**) and all survived (**Figure 2C**), as expected with this SARS-CoV-2 Omicron VoC (28).

To evaluate the effect of vaccination on SARS-CoV-2 replication, on day 6 post-challenge all mice were sacrificed, and the lungs and NW were collected and processed for the presence of live infectious virus (**Figures 2D, E**), as well as for SARS-CoV-2 subgenomic E or genomic RdRp and ORF1 RNA (**Figures 2F, G**). No live infectious virus was detected in the lungs of mice immunized with MVA-S(3P), MVA-S(3Pbeta), MVA-S(3P_BA.1) or the bivalent combination, whereas significantly higher viral yields were detected in the lungs of the MVA-WT control group (**Figure 2D**). On the other hand, no live infectious virus was detected in NW samples obtained from all mice, including the MVA-WT control group (**Figure 2E**), suggesting that, at this time point, the virus was naturally cleared from the nasal cavity of all mice.

In line with the results of live infectious virus yields, SARS-CoV-2 subgenomic and genomic RNA levels were significantly lower in the lungs of mice immunized with MVA-S(3P), MVA-S(3Pbeta), MVA-S(3P_BA.1), or the bivalent combination than in the MVA-WT control group (**Figure 2F**). Mice vaccinated with MVA-S(3Pbeta) or with the bivalent combination of MVA-S(3P) with MVA-S(3P_BA.1) exhibited the lowest levels of SARS-CoV-2 subgenomic and genomic RNA, while mice immunized with MVA-S(3P) have significantly higher levels of SARS-CoV-2 RNA than the other vaccinated groups (**Figure 2F**). In NW, no significant differences in SARS-CoV-2 subgenomic (E) and genomic (RdRp) RNA levels were observed between groups (**Figure 2G**), suggesting that IM vaccination did not impair viral replication in the nasal cavity at this time point.

**Figure 3:**
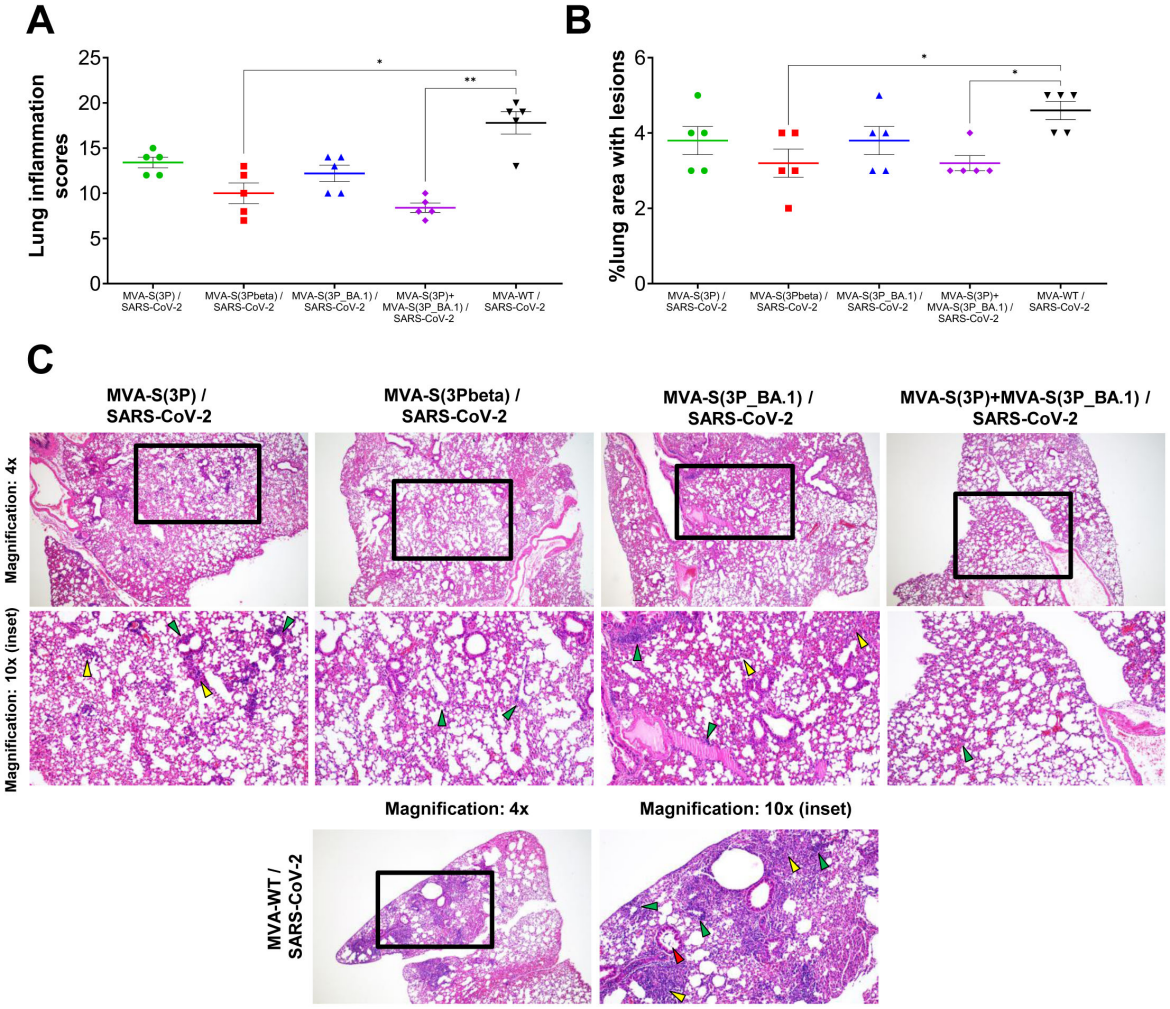
MVA-based vaccine candidates expressing S protein from different SARS-CoV-2 variants reduce lung pathology in K18-hACE2 transgenic mice challenged with SARS-CoV-2 Omicron (B.1.1.529) variant. **(A)** Mean and SEM of cumulative histopathological lesion scores in lung samples taken from immunized K18-hACE2 mice euthanized at day 6 post-challenge. Unpaired nonparametric Mann-Whitney test: *p < 0.033; **p < 0.002. **(B)** Percentage of lung area affected by inflammatory lesions in lung samples taken from immunized K18-hACE2 mice euthanized at day 6 post-challenge. Ordinary one-way ANOVA followed by Tukey’s multiple comparisons test: *p < 0.033. **(C)** Representative lung histopathological sections (haematoxylin & eosin staining) observed in immunized K18-hACE2 transgenic mice euthanized at day 6 post-challenge. A general view of the lung area (magnification: 4x) along with histopathological details from selected lung areas (black boxes) have been displayed (magnification: 10x). In mice immunized with MVA-based vaccine candidates expressing S from different SARS-CoV-2 variants, alveolar spaces were larger and more evident, while inflammatory changes were less severe than those observed in mice immunized with MVA-WT (unprotected control group). Mice immunized with MVA-based vaccine candidates expressing S from different SARS-CoV-2 variants showed mild lung lesions characterized by the presence of mild to moderate septal thickening, multifocal mild perivascular and peribronchiolar mononuclear infiltrates (green arrowheads) consisting mainly of lymphocytes, as well as occasional alveoli with mild cellular infiltrates (yellow arrowheads). In contrast, in mice immunized with MVA-WT inflammatory lesions were more severe and diffuse. Such lesions were characterized by diffuse moderate to severe septal thickening, disseminated severe perivascular and peribronchiolar mononuclear infiltrates (green arrowheads), alveolar spaces densely populated by inflammatory cells (yellow arrowheads), mainly monocytes, and bronchi or bronchioles (red arrowheads) with detached epithelium or inflammatory cells in the lumen (bronchitis/bronchiolitis).

Lung histopathological analysis at day 6 post-challenge showed that all vaccinated mice presented lesser lung inflammation scores (**Figure 3A**) and minor percentages of lung areas with lesions (**Figure 3B**) than MVA-WT control mice. This reduction was significant in mice vaccinated with MVA-S(3Pbeta) or with the bivalent combination (**Figures 3A, B**), following a pattern similar to that observed in the evaluation of SARS-CoV-2 RNA in the lungs (**Figure 2F**). Representative images of lung sections illustrate that mice vaccinated with MVA-S(3P), MVA-S(3Pbeta), MVA-S(3P_BA.1), or the bivalent combination only displayed focal thickening of the alveolar septa, occasional mild perivascular and peribronchiolar mononuclear infiltrates and the sporadic appearance of mild inflammatory infiltrates within the alveoli, while mice inoculated with control MVA-WT exhibited diffuse moderate to severe thickening of the alveolar septa, diffuse severe mononuclear cell infiltrates within the alveolar spaces, and disseminated severe perivascular and peribronchiolar mononuclear infiltrates (**Figure 3C**).

**Figure 4:**
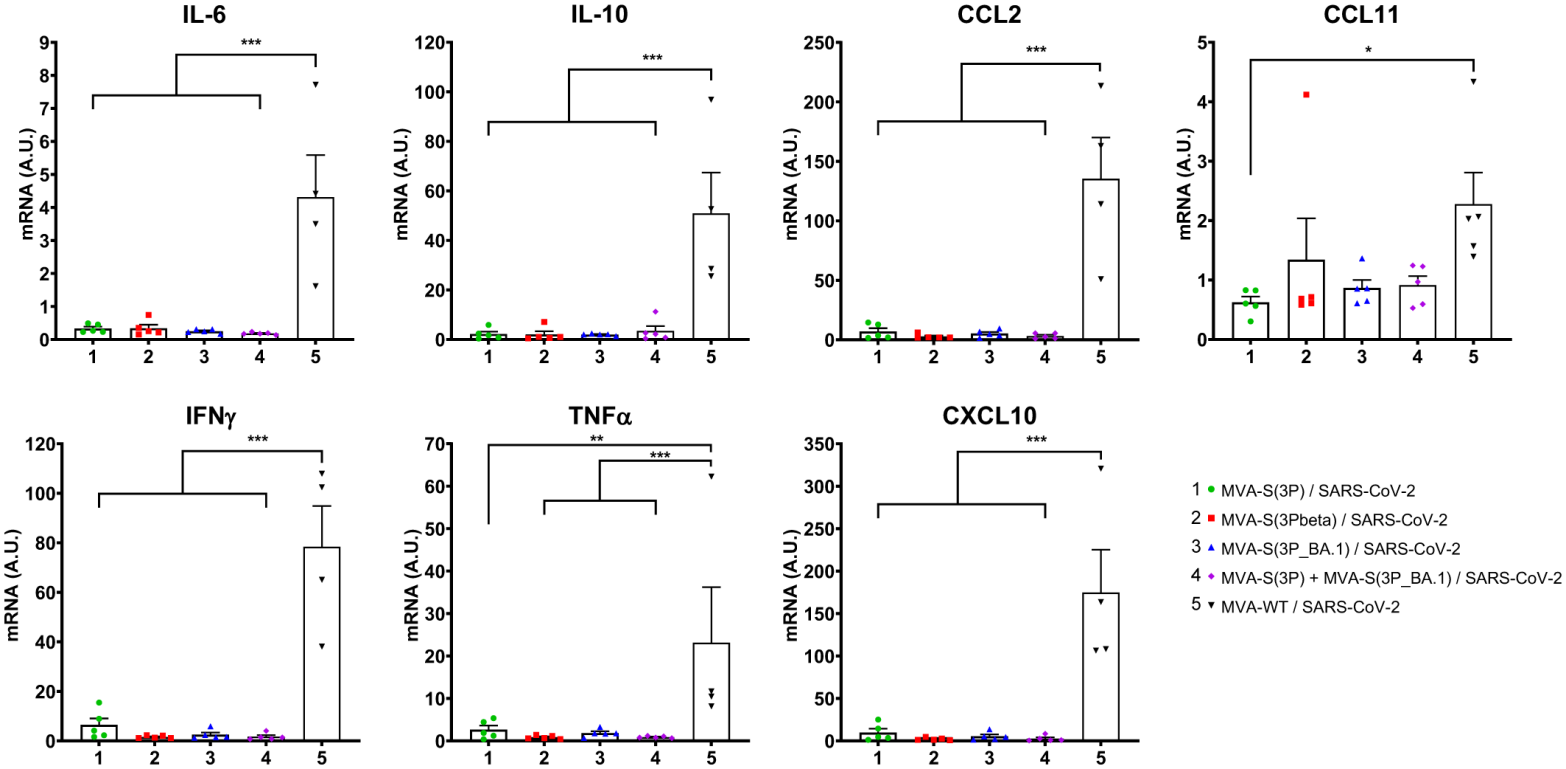
Vaccination with MVA-based vaccine candidates expressing S protein from different SARS-CoV-2 variants diminish levels of proinflammatory cytokines in K18-hACE2 transgenic mice challenged with SARS-CoV-2 Omicron (B.1.1.529) variant. mRNA levels of several cytokines/chemokines were detected by RT-qPCR in lungs obtained at 6 days post-challenge. Mean RNA levels (in A.U. normalized to uninfected mice) from duplicates of each sample and SEM of each group are represented. Ordinary one-way ANOVA of transformed data followed by Tukey’s multiple comparisons test: *p < 0.033; **p < 0.002; ***p < 0.0002.

Moreover, the impact of vaccination on the pro-inflammatory cytokine profile induced in the lungs of infected mice was analyzed at day 6 post-challenge by measuring by RT-qPCR in lung samples the mRNA levels of key cytokines (**Figure 4**). The results showed that vaccination with MVA-S(3P), MVA-S(3Pbeta), MVA-S(3P_BA.1), or the bivalent combination significantly downregulated the mRNA levels of IL-6, IL-10, CCL2, CCL11, IFNγ, TNFα and CXCL10, compared to the MVA-WT control group (**Figure 4**).

### Vaccination of K18-hACE2 transgenic mice with MVA-S(3P), MVA-S(3Pbeta), MVA-S(3P_BA.1), or a bivalent MVA-S(3P)/MVA-S(3P_BA.1) combination induced potent and broadly cross-reactive serum antibody responses against SARS-CoV-2

**Figure 5:**
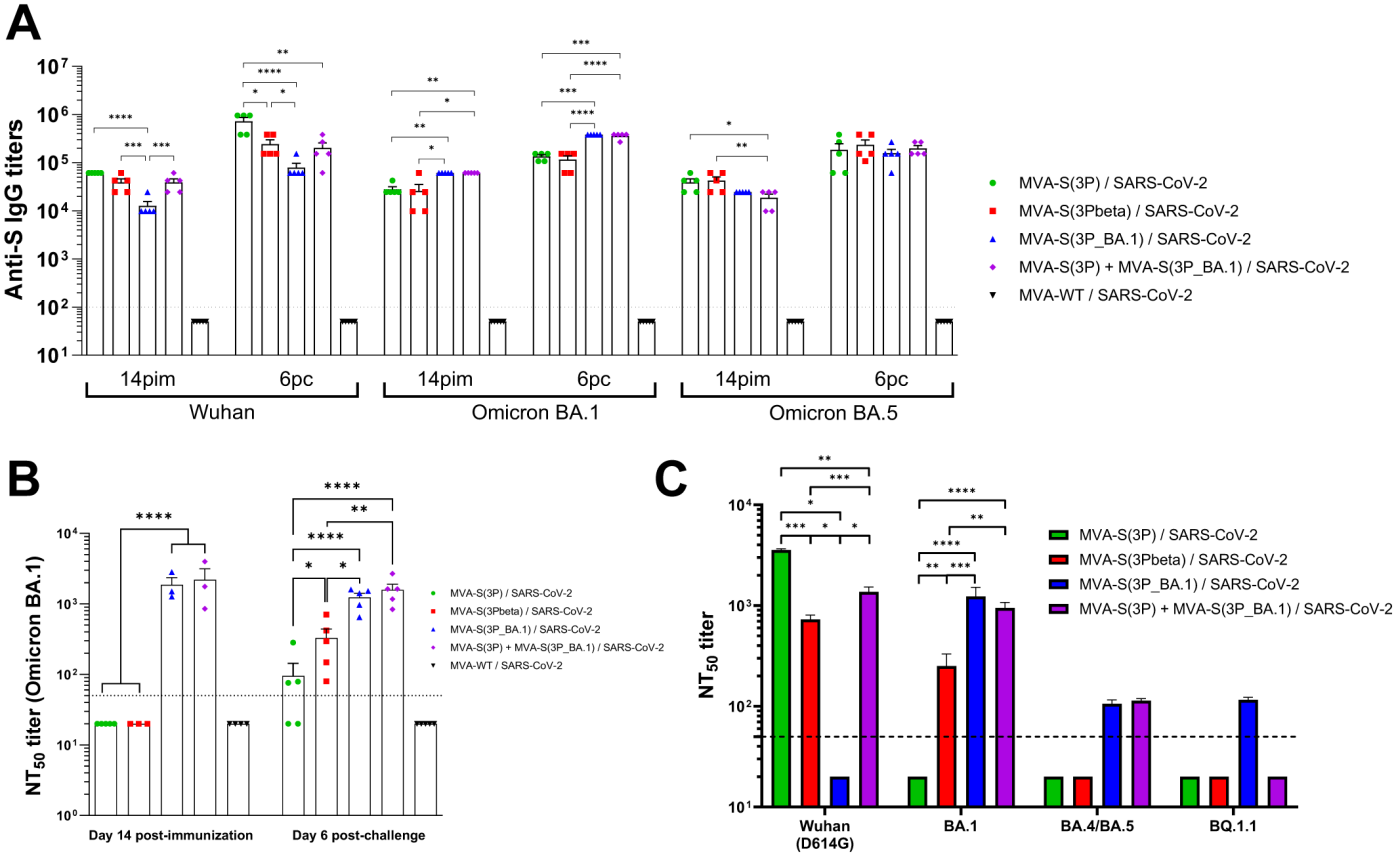
Vaccination with MVA-based vaccine candidates expressing S protein from different SARS-CoV-2 variants induce SARS-CoV-2-specific humoral immune responses in Omicron-challenged K18-hACE2 transgenic mice. **(A)** Anti-S IgG titers against Wuhan, Omicron BA.1 and Omicron BA.5 determined by ELISA in individual mouse serum samples collected from K18-hACE2 mice at day 14 post-immunization (pi; pre-challenge), and 6 post-challenge (pc). Mean endpoint titers of each sample from duplicates and SEM from each group are represented. Dashed line represents the limit of detection. Unpaired ordinary one-way ANOVA followed by Tukey’s multiple comparisons test of transformed data: *p < 0.033; **p < 0.002; ***p < 0.0002; ****p<0.0001. **(B)** Neutralizing antibody titers against SARS-CoV-2 Omicron BA.1. NT_50_ titers were evaluated in individual mouse serum samples collected at day 14 post-immunization (pre-challenge), and 6 post-challenge using a live virus MNT assay. Mean NT_50_ values of each sample from triplicates and SEM from each group are represented. Dotted line represented the limit of detection. Unpaired ordinary one-way ANOVA followed by Tukey’s multiple comparisons test of transformed data: *p < 0.033; **p < 0.002; ****p<0.0001. **(C)** SARS-CoV-2 neutralizing antibody titers against Wuhan (containing the D614 mutation in the S protein), Omicron BA.1, BA.4/BA.5, and BQ.1.1. NT_50_ titers were evaluated in pooled mouse serum samples collected at day 14 post-immunization, using VSV-based pseudoparticles expressing the SARS-CoV-2 S protein of Wuhan and of different Omicron subvariants. Mean NT_50_ values and 95% confidence intervals from triplicates of each pooled group sample are represented. The dashed line represents the limit of detection. Brown-Forsythe and Welch ANOVA test of transformed data: *p < 0.033; **p < 0.002; ***p < 0.0002; ****p<0.0001.

Since humoral immunity, and specifically neutralizing antibodies, is the main correlate of protection against SARS-CoV-2 infection (29), we next analyzed the presence of antibody responses against different SARS-CoV-2 variants in serum samples from transgenic K18-hACE2 mice vaccinated with MVA-S(3P), MVA-S(3Pbeta), MVA-S(3P_BA.1), or the bivalent vaccine candidate MVA-S(3P)/MVA-S(3P_BA.1) (**Figure 5**).

Measurement by ELISA, on days 14 post-immunization (pre-challenge) and 6 post-challenge, of total binding IgG antibodies against Wuhan, Omicron BA.1 or BA.5 S proteins, revealed a potent induction of binding antibodies in all vaccinated groups, except in the MVA-WT control group, with endpoint IgG titers between 10^4^-10^5^ on day 14 post-immunization, which enhanced to titers between 10^5^-10^6^ on day 6 post-challenge (**Figure 5A**), indicating that none of the groups induced sterile immunity. Interestingly, vaccination with MVA-S(3P) induced, at both time points analyzed, significantly higher anti-S Wuhan IgG titers than vaccination with the other vaccine candidates, followed by the bivalent vaccine candidate and by MVA-S(3Pbeta), being MVA-S(3P_BA.1) the vaccine candidate that elicited lower levels of binding IgG antibodies against the ancestral Wuhan strain (**Figure 5A**). On the other hand, vaccination with MVA-S(3P_BA.1), or with the bivalent MVA-S(3P)/MVA-S(3P_BA.1) vaccine candidate induced, both on day 14 post-immunization and on day 6 post-challenge, significantly higher anti-S Omicron BA.1 IgG titers than vaccination with MVA-S(3P) or MVA-S(3Pbeta) (**Figure 5A**). Regarding IgG titers against a mismatch Omicron subvariant, such as BA.5, mice vaccinated with MVA-S(3P) and MVA-S(3Pbeta) exhibited slightly higher binding anti-S IgG titers than those vaccinated with MVA-S(3P_BA.1) or the bivalent vaccine candidate on day 14 post-immunization, but these small differences between groups disappeared on day 6 post-challenge (**Figure 5A**).

Analysis of the levels of neutralizing antibodies against the live Omicron BA.1 variant revealed that, on day 14 post-immunization, only mice vaccinated with MVA-S(3P_BA.1) or the bivalent MVA-S(3P)/MVA-S(3P_BA.1) vaccine candidate elicited detectable and similar NT_50_ titers in serum samples (**Figure 5B**). On day 6 post-challenge, NT_50_ titers against live Omicron BA.1 in the serum of mice vaccinated with MVA-S(3P) and MVA-S(3Pbeta) scarcely raised, with the serum from MVA-S(3Pbeta)-vaccinated mice showing significant higher levels of neutralizing antibodies than in MVA-S(3P)-vaccinated animals (**Figure 5B**). On the other hand, neutralizing antibody titers against live Omicron BA.1 in mice vaccinated with MVA-S(3P_BA.1) or the bivalent vaccine candidate remained at the same level as they were on day 14 post-immunization, staying significantly higher than those of mice vaccinated with the other two vaccine candidates (**Figure 5B**).

Furthermore, we analyzed in pooled sera obtained on day 14 post-immunization the titers of neutralizing antibodies against the ancestral Wuhan strain (an isolate containing the D614G mutation in the S protein) and several Omicron subvariants (BA.1, BA.4/BA.5, and BQ.1.1) by using SARS-CoV-2 pseudotyped VSVs (**Figure 5C**). The results showed that vaccination with MVA-S(3P), MVA-S(3Pbeta) and the bivalent MVA-S(3P)/MVA-S(3P_BA.1) vaccine candidate elicited high NT_50_ titers against the ancestral Wuhan D614G SARS-CoV-2 (with MVA-S(3P) inducing significantly higher NT_50_ titers), while MVA-S(3P_BA.1) did not induce detectable levels of neutralizing antibodies against the ancestral Wuhan D614G SARS-CoV-2 (**Figure 5C**). On the other hand, MVA-S(3P_BA.1) and the bivalent vaccine candidate elicited high NT_50_ titers against the Omicron BA.1 variant, while MVA-S(3Pbeta) induced lower titers and MVA-S(3P) did not induce detectable NT_50_ titers. Finally, MVA-S(3P_BA.1) and the bivalent vaccine candidate elicited low but similar NT_50_ titers against Omicron BA.4/5, whereas the only MVA-S(3P_BA.1) induced detectable NT_50_ titers against the BQ.1.1 subvariant (**Figure 5C**).

### Intranasal immunization of C57BL/6 mice with MVA-S(3P), MVA-S(3Pbeta), MVA-S(3P_BA.1), or a bivalent MVA-S(3P)/MVA-S(3P_BA.1) combination induced systemic and local SARS-CoV-2 Omicron-specific CD4^+^ and CD8^+^ T-cellular and humoral immune responses

**Figure 6:**
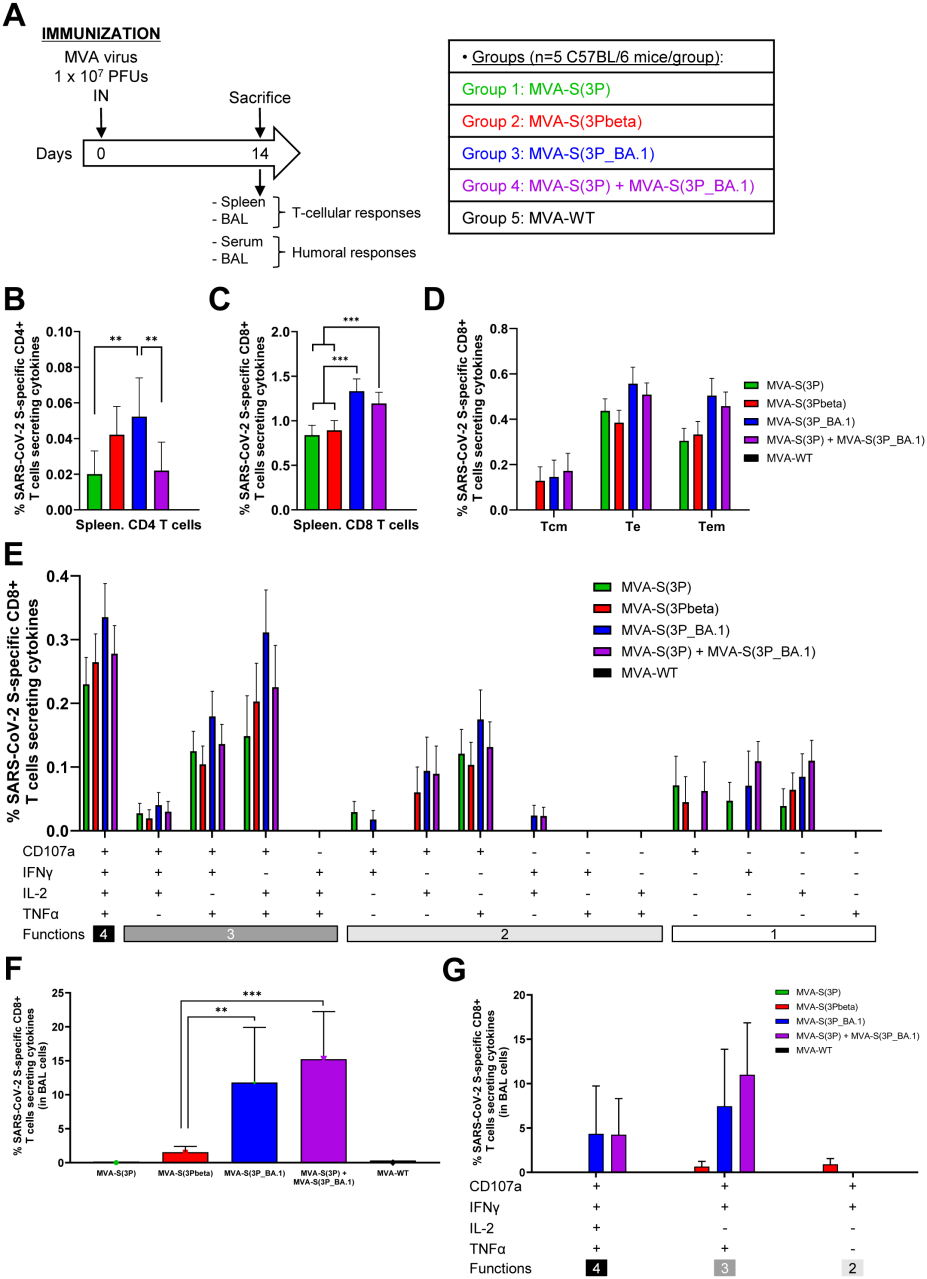
SARS-CoV-2-specific immunogenicity in C57BL/6 mice immunized with one IN dose of MVA-based vaccine candidates expressing S protein from different SARS-CoV-2 variants. **(A)** Immunogenicity study schedule. Groups of female C57BL/6 mice (n=5 per group; 6 to 8 weeks-old) were slightly anesthetized and each mouse received one dose of 1 x 10^7^ PFUs of MVA-based vaccine candidates expressing S protein from different SARS-CoV-2 variants by the IN route in 50 μl of PBS, as indicated. Mice inoculated with non-recombinant MVA-WT were used as a control group. At day 14 after the immunization, mice were euthanized and serum, spleens and BAL samples from each mouse were collected to evaluate SARS-CoV-2-specific T-cellular and humoral immune responses. **(B-G)** SARS-CoV-2 S-specific T-cellular immune responses, directed against a mixture of S1 and S2 Omicron (B.1.1.529) peptide pools, were analyzed in pools of splenocytes **(B-E)**, and pools of BAL cells **(F-G)** by ICS, as described in Materials and Methods. **(B, C)** Magnitude of Omicron (B.1.1.529) S-specific CD4^+^ **(B)** and CD8^+^ **(C)** T-cell immune responses in spleens. Percentages of CD4^+^ or CD8^+^ T cells expressing CD107a and/or producing IFNγ and/or TNFα and/or IL-2. **(D)** Memory phenotypic profiles of the SARS-CoV-2 S-specific CD8^+^ T cells in spleens. Percentages of T central memory (Tcm) (CD127^+^/CD62L^+^), T effector (Te) (CD127^−^/CD62L^−^) and T effector memory (Tem) (CD127^+^/CD62L^−^) SARS-CoV-2 Omicron (B.1.1.529) S-specific CD8^+^ T cells expressing CD107a and/or producing IFNγ and/or TNFα and/or IL-2. **(E)** Polyfunctional profile (based on expression of selected markers CD107a, IFNγ, TNFα, and IL-2) of total SARS-CoV-2 Omicron (B.1.1.529) S-specific CD8^+^ T cells in spleens. The response profiles are shown on the x axis, and the percentages of T cells for each of the vaccinated groups are shown on the y axis. **(F)** Magnitude of Omicron (B.1.1.529) S-specific CD8^+^ T-cells in BAL cells. Percentage of CD8^+^ T cells expressing CD107a and/or producing IFNγ and/or TNFα and/or IL-2. **(G)** Polyfunctional profile (based on expression of selected markers CD107a, IFNγ, TNFα, and IL-2) of total SARS-CoV-2 Omicron (B.1.1.529) S-specific CD8^+^ T cells in BAL cells. P values were determined as described in Materials and Methods using an approach that corrects measurements for the medium response, calculating confidence intervals (**, P < 0.005; ***, P < 0.001).

Both, local and systemic, immune responses are determining factors in the effectiveness of protection against SARS-CoV-2 (29). We have previously shown that MVA-S(3P) is highly immunogenic in these two compartments, when administered via the mucosal IN route, activating SARS-CoV-2-specific T-cellular and humoral immune responses (12, 13). Thus, we next evaluated SARS-CoV-2-specific T-cell and humoral immunogenicity induced in C57BL/6 mice (n=6/group) after a single IN dose of 1 x 10^7^ PFUs of MVA-S(3P), MVA-S(3Pbeta), MVA-S(3P_BA.1), bivalent MVA-S(3P)/MVA-S(3P_BA.1) vaccine candidate, or MVA-WT (as a negative control group). Mice were euthanized at 14 days post-immunization and SARS-CoV-2 S-specific T-cell immune responses were evaluated systemically, in spleen, or locally, in BAL; while SARS-CoV-2-specific humoral responses were evaluated in serum and BAL samples (**Figure 6A**).

Regarding SARS-CoV-2 S-specific T-cell immune responses, splenocytes or BAL cells were stimulated *ex vivo* with a mixture of Omicron (B.1.1.529) S peptide pools (spanning the entire S protein) and an ICS assay was performed to measure the induction of SARS-CoV-2 Omicron S-specific CD4^+^ and CD8^+^ T cells expressing CD107a, and/or secreting IFNγ, and/or TNFα, and/or IL-2. The results showed that vaccinated mice elicited an overall S-specific cellular response mainly mediated by CD8^+^ T lymphocytes of a higher magnitude in the local compartment, BAL, than in the spleen (**Figures 6B–G**). In spleen, MVA-S(3P_BA.1)-vaccinated mice elicited significantly higher Omicron S-specific CD4^+^ T-cell immune responses than MVA-S(3P) and the MVA-S(3P)/MVA-S(3P_BA.1) bivalent vaccine candidate (**Figure 6B**). Regarding CD8^+^ T-cell responses, mice vaccinated with MVA-S(3P_BA.1) and the bivalent vaccine candidate induced a significantly higher magnitude of Omicron S-specific CD8^+^ T cells than mice vaccinated with MVA-S(3P) or MVA-S(3Pbeta) (**Figure 6C**). Omicron S-specific CD8^+^ T-cell immune responses elicited in all four vaccinated groups were mainly of a T effector (Te) or T effector memory (Tem) phenotype (**Figure 6D**), and exhibited a highly polyfunctional profile, with the majority of Omicron S-specific CD8^+^ T cells displaying four (CD107a-IFNγ-TNFα-IL-2) or three (CD107a-TNFα-IL-2, CD107a-IFNγ-TNFα) functions (**Figure 6E**). In BAL cells, MVA-S(3P_BA.1) and the bivalent vaccine candidate induced a significantly higher magnitude of Omicron S-specific CD8^+^ T cells than MVA-S(3P) or MVA-S(3Pbeta) (**Figure 6F**), with no S-specific CD4^+^ T cells detected in BAL cells from any vaccinated group (data not shown). Furthermore, Omicron S-specific CD8^+^ T cells present in BAL cells also displayed a highly polyfunctional profile with cells mainly exhibiting four (CD107a-IFNγ-TNFα-IL-2) or three (CD107a-IFNγ-TNFα) functions (**Figure 6G**).

**Figure 7:**
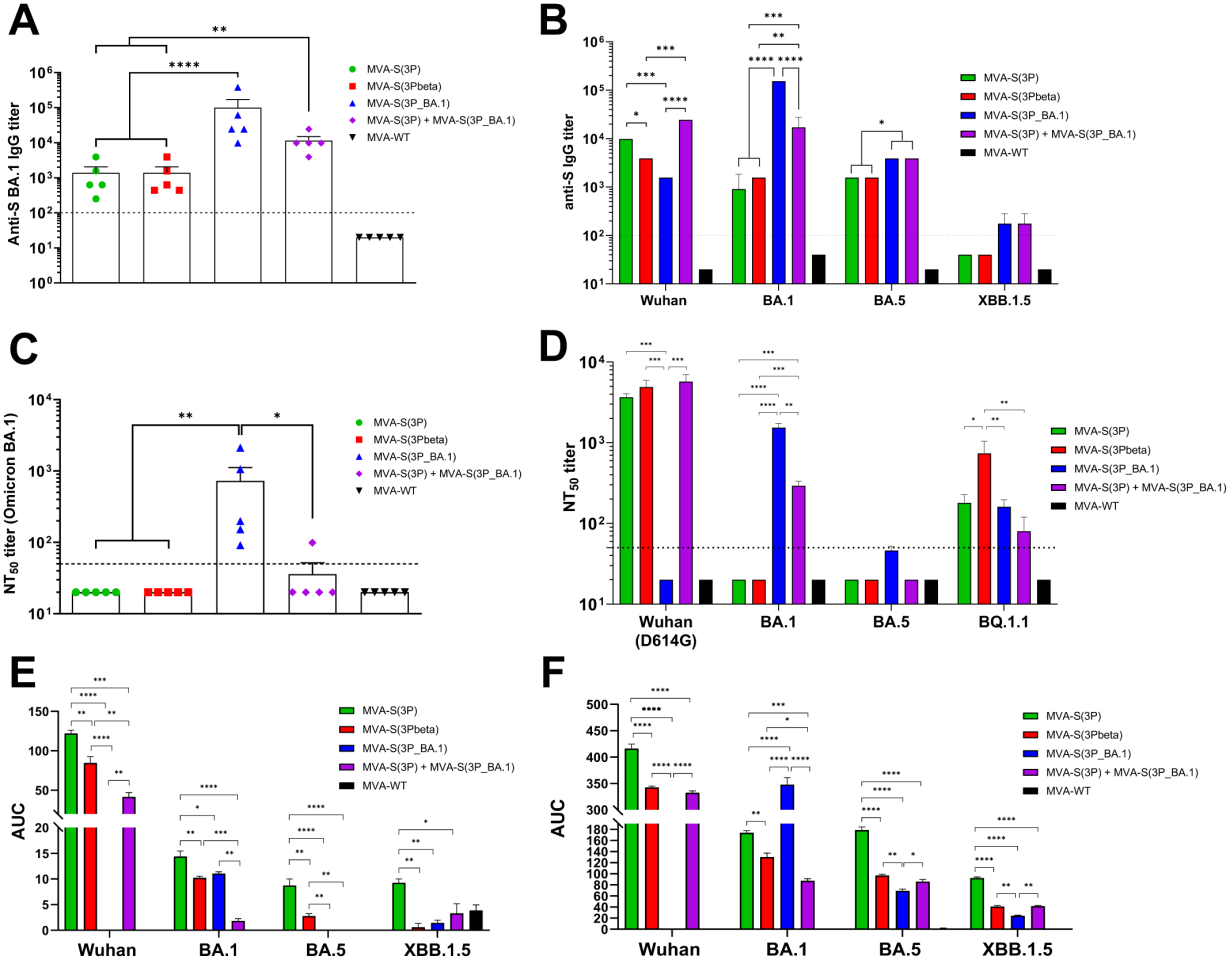
SARS-CoV-2-specific humoral immune responses elicited in C57BL/6 mice immunized with one IN dose of MVA-based vaccine candidates expressing S protein from different SARS-CoV-2 variants. Immunization schedule is indicated in **Figure 6A**, and SARS-CoV-2-specific humoral immune responses were evaluated in serum and BAL samples obtained at 14 days post-immunization. **(A)** Binding IgG titers against Omicron BA.1 S protein determined by ELISA in individual mouse serum samples. Mean endpoint titers of each sample from duplicates and SEM from each group are represented. Dashed line represents the limit of detection. Unpaired ordinary one-way ANOVA followed by Tukey’s multiple comparisons test of transformed data: **p < 0.002; ****p<0.0001. **(B)** Binding IgG titers against S protein from Wuhan, Omicron BA.1, BA.5 and XBB.1.5 determined by ELISA in pooled mouse serum samples. Mean endpoint titers of each sample from duplicates and SEM are represented. Dashed line represents the limit of detection. Ordinary two-way ANOVA followed by Tukey’s multiple comparisons test, with a single pool variance: *p < 0.033; **p < 0.002; ***p < 0.0002; ****p<0.0001. **(C)** Neutralizing antibody titers against SARS-CoV-2 Omicron BA.1. SARS-CoV-2 NT_50_ antibody titers against Omicron (B.1.1.529) BA.1 variant determined in individual mouse serum samples by using a live virus MNT assay. Mean NT_50_ values of each sample from triplicates and SEM from each group are represented. Dashed line represents the limit of detection. Unpaired ordinary one-way ANOVA followed by Tukey’s multiple comparisons test of transformed data: *p < 0.033; **p < 0.002. **(D)** SARS-CoV-2 neutralizing antibody titers against Wuhan (MAD6 isolate, containing the D614 mutation in the S protein), Omicron BA.1, BA.4/BA.5, and BQ.1.1. NT_50_ titers against live SARS-CoV-2 Wuhan (MAD6 isolate), Omicron BA.1, BA.5 and BQ.1.1 were evaluated in pooled mouse serum samples, by using a live virus MNT assay. Mean NT_50_ values and 95% confidence intervals from triplicates of each pooled group sample are represented. The dashed line represents the limit of detection. Brown-Forsythe and Welch ANOVA test of transformed data: *p < 0.033; **p < 0.002; ***p < 0.0002; ****p<0.0001. **(E, F)** Anti-S IgA **(E)** and IgG **(F)** antibody levels against Wuhan, Omicron BA.1, BA.5 and XBB.1.5 determined by ELISA in pooled BAL samples, analyzed in duplicate. Area under the curve (AUC) mean values and SEM are represented. Unpaired ordinary one-way ANOVA followed by Tukey’s multiple comparisons test *p < 0.033; **p < 0.002; ***p < 0.0002; ****p<0.0001.

Regarding SARS-CoV-2-specific humoral responses, the results showed that in individual serum samples, mice vaccinated with MVA-S(3P_BA.1) or the bivalent MVA-S(3P)/MVA-S(3P_BA.1) vaccine candidate induced similar and significantly higher titers of total IgG antibodies against Omicron BA.1 S protein than mice vaccinated with MVA-S(3P) or MVA-S(3Pbeta) (**Figure 7A**). Moreover, comparative analysis of total binding IgG antibody titers against S protein from other SARS-CoV-2 variants was performed in pooled sera (**Figure 7B**). Mice vaccinated with MVA-S(3P) or the bivalent vaccine candidate elicited the highest titers of total binding IgG antibodies against Wuhan S protein, whereas MVA-S(3P_BA.1) and the bivalent vaccine candidate induced significantly the greatest titers of total binding IgG against BA.1 and BA.5 S proteins (**Figure 7B**). Only mice vaccinated with MVA-S(3P_BA.1) or the bivalent vaccine candidate prompted detectable anti-S XBB.1.5 total IgG titers (**Figure 7B**).

Concerning the neutralization capacity against live Omicron BA.1, assayed in individual serum samples, only IN vaccination with MVA-S(3P_BA.1) induced detectable anti-Omicron BA.1 NT_50_ titers (**Figure 7C**). The analysis in pooled sera of the NT_50_ titers against other live SARS-CoV-2 variants showed that mice vaccinated with MVA-S(3P), MVA-S(3Pbeta) and the bivalent vaccine candidate elicited high NT_50_ titers against the ancestral Wuhan SARS-CoV-2 (MAD6 isolate), whereas no detectable NT_50_ titers were found in sera from mice immunized with MVA-S(3P_BA.1) (**Figure 7D**). On the other hand, only mice vaccinated with MVA-S(3P_BA.1) and the bivalent vaccine candidate induced NT_50_ titers against Omicron BA.1, while none of the sera from the different vaccinated groups elicited detectable NT_50_ titers against the Omicron BA.5 subvariant (**Figure 7D**). Lastly, sera from all vaccinated groups elicited detectable NT_50_ titers against the Omicron BQ.1.1 subvariant, with MVA-S(3Pbeta) being the vaccine candidate inducing significantly higher NT_50_ titers (**Figure 7D**).

Finally, we evaluated the capacity of the different MVA-based vaccine candidates to elicit local mucosal humoral immune responses against SARS-CoV-2 in BAL samples by using an ELISA analysis of S-specific binding IgA (**Figure 7E**) and IgG (**Figure 7F**) antibodies. The results showed that both IgA and IgG antibodies were detected against different S variants, in pooled BAL samples from all vaccinated groups. In particular, mice vaccinated with MVA-S(3P) elicited the highest levels of anti-S IgA to Wuhan, as expected, but surprisingly also against S protein from BA.1, BA.5 and XBB.1.5 (**Figure 7E**). Vaccination with MVA-S(3P_BA.1) only elicited detectable IgA levels against S protein from BA.1, while the bivalent vaccine candidate induced IgA antibodies against S protein from Wuhan and BA.1 (**Figure 7E**). Regarding IgG antibodies, IN vaccination with MVA-S(3P_BA.1) elicited significantly higher IgG levels against Omicron BA.1 S protein than the other vaccine candidates, with also an induction of IgG antibodies against Omicron BA.5 and XBB.1.5, but undetectable levels against Wuhan S protein (**Figure 7F**). On the other hand, MVA-S(3P) induced higher IgG levels against Wuhan S protein than the other vaccine candidates, but also induced IgG antibodies against S protein from BA.1, BA.5 and XBB.1.5 (**Figure 7F**). Importantly, the bivalent vaccine candidate induced IgG antibodies against S protein from all SARS-CoV-2 viruses tested (**Figure 7F**).

## Discussion

In late 2021, the emergence of the SARS-CoV-2 Omicron (B.1.1.529) variant raised concerns due to numerous mutations encounter in the viral S protein, suggesting an increased potential for transmission and the evasion of neutralizing antibodies (5, 7). Within a few weeks of its initial identification, this variant swiftly disseminated worldwide, displacing previously prevalent VoCs and triggering a resurgence in COVID-19 cases, even in populations with elevated levels of immunity from vaccinations and prior infections (3). Research indicates that Omicron (and now its subvariants) exhibit reduced susceptibility to protective antibodies generated by past infections and by approved vaccines designed for earlier SARS-CoV-2 lineages (6). Thus, the capacity of Omicron to spread, even among vaccinated individuals, has spurred a global call for updated vaccine regimens capable of providing effective protection against Omicron, and its newer subvariants (6).

To address the question on how a vaccine against Omicron compares head-to-head with vaccines against other variants, this report highlights the effectiveness in mice against lower respiratory tract infection and lung pathology of four MVA-based COVID-19 vaccine candidates expressing full-length prefusion-stabilized S proteins from Wuhan [MVA-S(3P)], Beta [MVA-S(3Pbeta)], or Omicron BA.1 [MVA-S(3P_BA.1)], as well as a bivalent vaccine combination composed of a 1:1 mixture of MVA-S(3P) and MVA-S(3P_BA.1). Therefore, in the efficacy experiments carried out in transgenic K18-hACE2 mice susceptible to SARS-CoV-2 infection, all these vaccine candidates demonstrated protective capabilities after SARS-CoV-2 Omicron infection, avoiding SARS-CoV-2 infectious viral loads, reducing viral mRNA, decreasing lung histopathological lesions and diminishing levels of proinflammatory cytokines in the lungs. These results complemented our previous observations in transgenic K18-hACE2 mice and Syrian hamsters that demonstrated in both animal models the potent efficacy of MVA-S(3P) to protect against the ancestral SARS-CoV-2 Wuhan strain (13, 14, 16), and also by the results in transgenic K18-hACE2 mice that showed protection of MVA-S(3P) and MVA-S(3Pbeta)-vaccinated animals against Beta VoC infection (12). Here, we observed that a single IM dose of any of the four MVA-based vaccine candidates, despite the expression of different S proteins from Wuhan, Beta and Omicron BA.1, provided complete protection against Omicron BA.1 infection, with undetectable SARS-CoV-2 Omicron viral loads in the lungs. This is of interest considering the undetectable or low BA.1-specific NT_50_ titers induced by MVA-S(3P) or MVA-S(3Pbeta). Similar results were found with synthetic multiantigen MVA-based vaccine candidates against the ancestral Wuhan SARS-CoV-2, Beta and Delta VoC, which despite eliciting strain-specific antibody responses, protected hamsters from body weight loss, lower respiratory tract infection and lung pathology following challenge with Omicron subvariants (30). Furthermore, passive transfer experiments showed that anti-Wuhan serum without detectable *in vitro* neutralizing activity for Omicron was partially protective *in vivo* in mice (31). However, in these two studies a two-dose regimen was followed, while in the present study we achieved protection against Omicron BA.1 using a single immunization with any of our four MVA-based vaccine candidates. While these findings may indicate that in transgenic K18-hACE2 mice only low neutralizing antibody titers are required to protect against Omicron infection, they may also indicate that other responses, besides neutralizing antibodies, contribute to the efficacy observed in this study. This may include humoral responses promoting Fc-mediated effector functions or T cell responses (29). Furthermore, the results from the study in K18-hACE2 mice provided compelling evidence that MVA-S(3P) and its variant-specific analogues, especially when combined, have significant potential to confer broad cross-immunity against several SARS-CoV-2 variants, including some of the most recent Omicron subvariants. This was evident in the analysis of total IgG titers elicited against the S protein from different variants in serum samples from K18-hACE2 mice.

While repeated booster vaccinations have shown efficacy in addressing declining effectiveness observed with initial Wuhan-based COVID-19 vaccines (32–34), there has been a development of variant-specific vaccines to enhance efficacy against emerging Omicron subvariants. Therefore, the European Medicines Agency has approved mRNA boosters with vaccines including BA.1-, BA.4/BA.5-, and XBB.1.5-specific modifications. Studies conducted in hamsters and non-human primates indicate that Wuhan-based COVID-19 vaccines can provide protection against Omicron B.1.1.529 and its BA.1 subvariant (35–38). However, the efficacy of Omicron-specific sequence modifications to improve COVID-19 vaccine efficacy is a subject of debate (39–41), with the pursuit of a pan-coronavirus vaccine. Our observations in transgenic K18-hACE2 mice demonstrating efficacy of MVA-S(3P) to protect against Omicron BA.1 lung infection supports that Wuhan-based COVID-19 vaccines have the capacity to confer cross-protective immunity against Omicron variant. Nevertheless, the BA.1-specific vaccine candidate MVA-S(3P_BA.1) and the bivalent vaccine candidate MVA-S(3P)/MVA-S(3P_BA.1) induced overall better Omicron-specific humoral and cellular immune responses compared to MVA-S(3P) in C57BL/6 mice. This improvement might correlate with the slightly enhanced efficacy in protecting against lower respiratory tract infection and lung pathology during the early phase (day 6 post-exposure) following homologous challenge with the Omicron variant in K18-hACE2 mice.

The immunogenicity experiment carried out in C57BL/6 mice demonstrated that a single IN immunization with MVA-S(3P_BA.1) or the bivalent MVA-S(3P)/MVA-S(3P_BA.1) vaccine candidate elicited not only comparable high BA.1-specific binding IgG and neutralizing antibody levels in serum samples, similarly to after a single IM vaccination in K18-hACE2 mice, but also local mucosal induction of anti-S BA.1 IgA and IgG antibodies. Although the potential role of mucosal IgA antibodies in protection against SARS-CoV-2 infection is still unknown, several studies suggest that S-specific mucosal IgA antibodies might confer some protection against respiratory tract infection (42, 43). Furthermore, these systemic and local humoral immune responses elicited by MVA-S(3P_BA.1) and the bivalent vaccine candidate MVA-S(3P)/MVA-S(3P_BA.1) were demonstrated to be broad-range, cross-reacting against other Omicron subvariants.

On the other hand, IN immunization with all our four MVA-based vaccine candidates expressing full-length prefusion-stabilized S proteins also induced local (in BAL cells) and systemic (in spleens) Omicron S-specific T-cell immune responses that were mainly mediated by CD8^+^ T cells and highly polyfunctional, with MVA-S(3P_BA.1) and the bivalent vaccine candidate MVA-S(3P)/MVA-S(3P_BA.1) inducing the highest magnitude. T cells are known to be less susceptible to antigen variation than antibodies and therefore considered a critical second line of defense to provide long-term protective immunity against SARS-CoV-2 that cross-recognize different VoCs, including Omicron (44–48). Our preclinical studies in mice also demonstrated the potent capacity of our initial Wuhan-based vaccine candidate MVA-S(3P) to stimulate S-specific T cells that can cross-react against a mismatched variant, such as Omicron (as revealed in this work), or Beta (12). This suggest that specific T-cell responses may be critically important for vaccine protection against SARS-CoV-2, especially when neutralizing antibody responses are suboptimal. Another captivating result observed in the present study is that our MVA-based vaccine candidates expressing full-length prefusion-stabilized S proteins largely induced Omicron S-specific T effector memory (Tem) cells. Circulating T central memory (Tcm) and Tem cells are developed in naturally infected individuals and retain their ability to proliferate upon stimulation, enabling them to activate upon reinfections to ensure rapid clearance and a better disease course (47, 49, 50).

We recognize some limitations in our research: i) Since the Omicron (B.1.1.529) variant of SARS-CoV-2 multiplies faster but to a lesser extent in lung cells, the timing of our analysis may not have been the ideal; ii) while we assessed protection against viral infection and disease, we did not examine the impact on virus transmission, which remains to be explored; iii) our assessment of immune responses and protection was performed shortly after vaccination. To determine the longevity of both mucosal and systemic immune responses, analysis will need to be performed at later time points; iv) our investigations with nasally administered MVA vaccines were limited to a single-dose regimen. More studies are needed to determine its effectiveness as a booster or in the context of hybrid immunity after natural infection; v) despite observing strong T cell responses following intranasal vaccination with MVA-vectored vaccines, the specific role of these T cells in providing protection was not conclusively identified. Future research should involve immunization of CD8^+^ T cell-deficient mice, removal of CD4^+^ or CD8^+^ T cells from the airways, lungs, or bloodstream, or use of adoptive transfer of immune T cells; vi) our experiments were performed in mice to allow rapid testing and comparisons between multiple groups. Validation of these results in other animal models such as hamsters or non-human primates is necessary to confirm their applicability; and vii) while our studies have preclinical value, their utility as SARS-CoV-2 vaccine needs to be confirmed in clinical trials.

Taken together, our results showed that protection against Omicron SARS-CoV-2 infection can be obtained with MVA vectors expressing S protein from either the parental Wuhan strain, the Beta VoC, the homologous Omicron VoC and a bivalent combination of both Wuhan plus Omicron. While the cross-reactive neutralizing antibodies vary between the vectors, protection correlated with diminished levels of virus replication in tissues, production of neutralizing antibodies and activation of T-cell responses, mainly CD8^+^ T cells. The enhanced immunogenicity induced by this Omicron vaccine analogue or by the bivalent vaccine candidate MVA-S(3P)/MVA-S(3P_BA.1) could potentially improve long-term immunogenicity and efficacy in previously vaccinated individuals, particularly against new subvariants. Moreover, the enhanced breadth of cross-immunity against multiple variants of SARS-CoV-2 elicited by the bivalent vaccine candidate MVA-S(3P)/MVA-S(3P_BA.1) represents a significant advantage and sets the stage for the development of multivalent MVA-based vaccines.

## Data Availability

The original contributions presented in the study are included in the article/supplementary material. Further inquiries can be directed to the corresponding authors.
